# A case of breast squamous cell carcinoma following breast augmentation with liquid silicone injection after 16 years

**DOI:** 10.1186/s40792-022-01378-w

**Published:** 2022-01-28

**Authors:** Ryosuke Toyonaka, Jun Ozeki, Yumi Koyama, Saaya Takahashi, Xiaoyan Tang, Hiroko Kobayashi, Maki Amano, Keiichiro Tada, Toshio Miki, Mayumi Tani

**Affiliations:** 1 Department of Digestive Surgery, School of Medicine, Nihon University, 30-1 Oyaguchi Kamicho, Itabashi-ku, Tokyo, Japan; 2 Division of Breast and Endocrine Surgery, Department of Surgery, Nihon University Hospital, 1-6 Kandasurugadai, Chiyoda-ku, Tokyo, Japan; 3 Division of Breast and Endocrine Surgery, Department of Surgery, Nihon University School of Medicine, 30-1 Oyaguchi Kamicho, Itabashi-ku, Tokyo, Japan; 4 Department of Pathology, Nihon University Hospital, 1-6 Kandasurugadai, Chiyoda-ku, Tokyo, Japan; 5 Department of Pathology, Nihon University School of Medicine, 30-1 Oyaguchi Kamicho, Itabashi-ku, Tokyo, Japan; 6 Department of Radiology, Nihon University Hospital, 1-6 Kandasurugadai, Chiyoda-ku, Tokyo, Japan; 7 Division of Biomedical Sciences, Department of Physiology, Nihon University School of Medicine, 30-1 Oyaguchi Kamicho, Itabashi-ku, Tokyo, Japan

**Keywords:** Breast augmentation, Liquid silicone injection, Squamous cell carcinoma, Breast neoplasm

## Abstract

**Background:**

Breast augmentation has been linked to various complications, including cancerous tumors. The majority type of breast cancer associated with breast augmentation is adenocarcinoma. Primary squamous cell carcinoma (SCC) of the breast is extremely rare in both augmented and non-augmented women. Due to the low incidence, the possible origin and the mechanism of carcinogenesis of the breast SCC are not well understood. Here, we report a rare case of pure SCC 16 years after breast augmentation with liquid silicone injection.

**Case presentation:**

A 51-year-old Japanese woman was suffered from prolonged breast fluid retention in her left breast. Multiple unknown foreign bodies caused difficulties to investigate the inflammatory focus with ultrasonography. After unsuccessful surgical drainage and antibiotics treatments, the long-standing fluid retention was surgically removed and pathologically investigated. SCC was found in the removed tissue, and the patient underwent a total left mastectomy followed by postoperative chemotherapy. Pathological analysis revealed multiple cystic structures with a hard shell which enclosed high viscous liquid. A qualitative analysis using a Fourier transform infrared spectroscope defined the liquid as pure silicon, which possibly caused the squamous cell carcinogenesis.

**Conclusions:**

Although liquid silicone injection is not a current option for breast augmentation, the injected silicone could result in cancerous tumor generation after years. This case revealed that unphysiological substances could lead to unexpected biological reactions, which caused difficulties in diagnosis with our routine examination. It will be required that accumulate information from more cases and develop novel diagnostic equipment and biomarkers to address these artificial substance-derived tumors.

## Background

Breast augmentation began with the injection method and has been improved with bag prostheses and cohesive bags. Surgeons have made efforts to reduce complications due to injected foreign bodies and leakage from breast implants. Numerous cases of breast cancer after breast augmentation due to injected foreign bodies have been reported [1–10]. Most of the implant-related breast cancers are adenocarcinomas, and squamous cell carcinomas are rarely reported [4, 5]. We report a case of pure squamous cell carcinoma of the breast after breast augmentation with liquid silicone.

## Case presentation

A 51-year-old Japanese woman noticed redness and swelling in her left breast 16 years after breast augmentation. In 2004, the patient had undergone bilateral breast augmentation in Korea. Although there was no information about the exact type of injected material or the quantity, the material was speculated as liquid silicone. The patient had a history of hepatitis C, but no family history of cancer including breast cancer. She had no traumatic breast injury history and no regular medications or allergies.

**Fig. 1:**
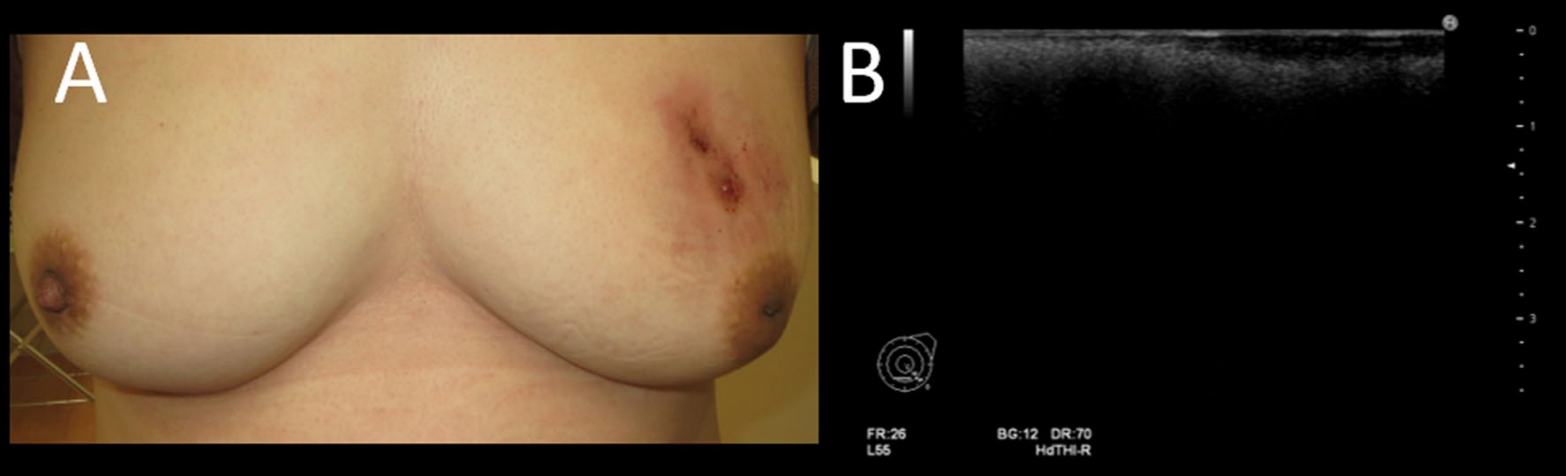
Skin findings and ultrasound images at initial examination. **A** Skin of the left breast was partially destructed and there was leakage of pus and serous fluid. **B** It was difficult to observe the depth due to strong backward echo caused by foreign body

Despite oral antibiotic therapy for suspected subcutaneous fluid retention, there was no improvement for 6 months. Palpation revealed redness, swelling, heat, and pain in the upper region of her left breast. The skin of the left breast was partially destructed and there was leakage of pus and serous fluid. Breast ultrasonography showed fluid retention in the left breast and enlarged reactive lymph nodes in the left axilla. The bilateral breasts were difficult to observe on ultrasonography due to foreign bodies (Fig. 1).

Contrast-enhanced computed tomography (CT) scans showed a large ulcerative lesion with thick enhancement on the ulcerative surface in the left breast. In addition, there were many nodules in the subcutaneous fat of both breasts, and some were encapsulated by calcification.

**Fig. 2:**
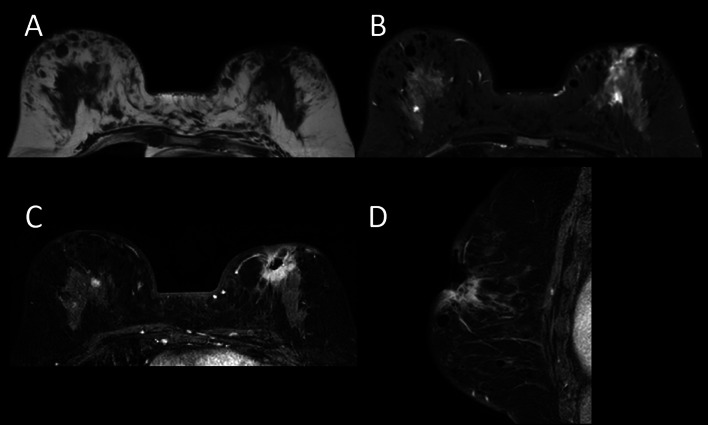
Breast MRI. **A** Axial T1WI **B** Axial fat suppressed T2WI **C** Axial contrast enhanced T1WI. **D** Sagittal contrast enhanced T1WI. Breast MRI shows a large ulcerative lesion in the left breast (arrow). There is an irregular shaped enhancing mass on the base of the ulcer and it is continuous with the mammary gland tissue (**C** and **D**). Many subcutaneous nodules are also seen in the both breasts. The T1-weighted image and fat-suppressed T2-weighted image (**B**) show no signal in those nodules (arrowhead). These are consistent with the liquid silicone materials injected before

Breast magnetic resonance imaging (MRI) shows a large ulcerative lesion in the left breast. There is an irregular-shaped enhancing mass on the base of the ulcer, and it is continuous with the mammary gland tissue. Many subcutaneous nodules are also seen in both breasts. The T1-weighted image and fat-suppressed T2-weighted image show no signal in those nodules. These are consistent with the liquid silicone materials injected before (Fig. 2).

Streptococcus pyogenes was detected in the culture of the subcutaneous fluid retention. Continuous drainage and following debridement were not effective. Thus, a partial left mastectomy was performed 101 days after the initial visit.

Pathology revealed a well-differentiated squamous cell carcinoma.

We performed a total left mastectomy and additional sentinel node biopsy.

An intraoperative sentinel lymph node evaluation was performed by one-step nucleic acid amplification (OSNA) assay. Due to the positive result of the OSNA assay, we performed an additional axillary lymph node dissection. Pathological examination revealed no metastasis in the dissected axillary nodes, and the resection margins were negative. After the surgery, the patient received four cycles of epirubicin, cyclophosphamide therapy, and four cycles of docetaxel therapy.

### Histopathological findings

**Fig. 3:**
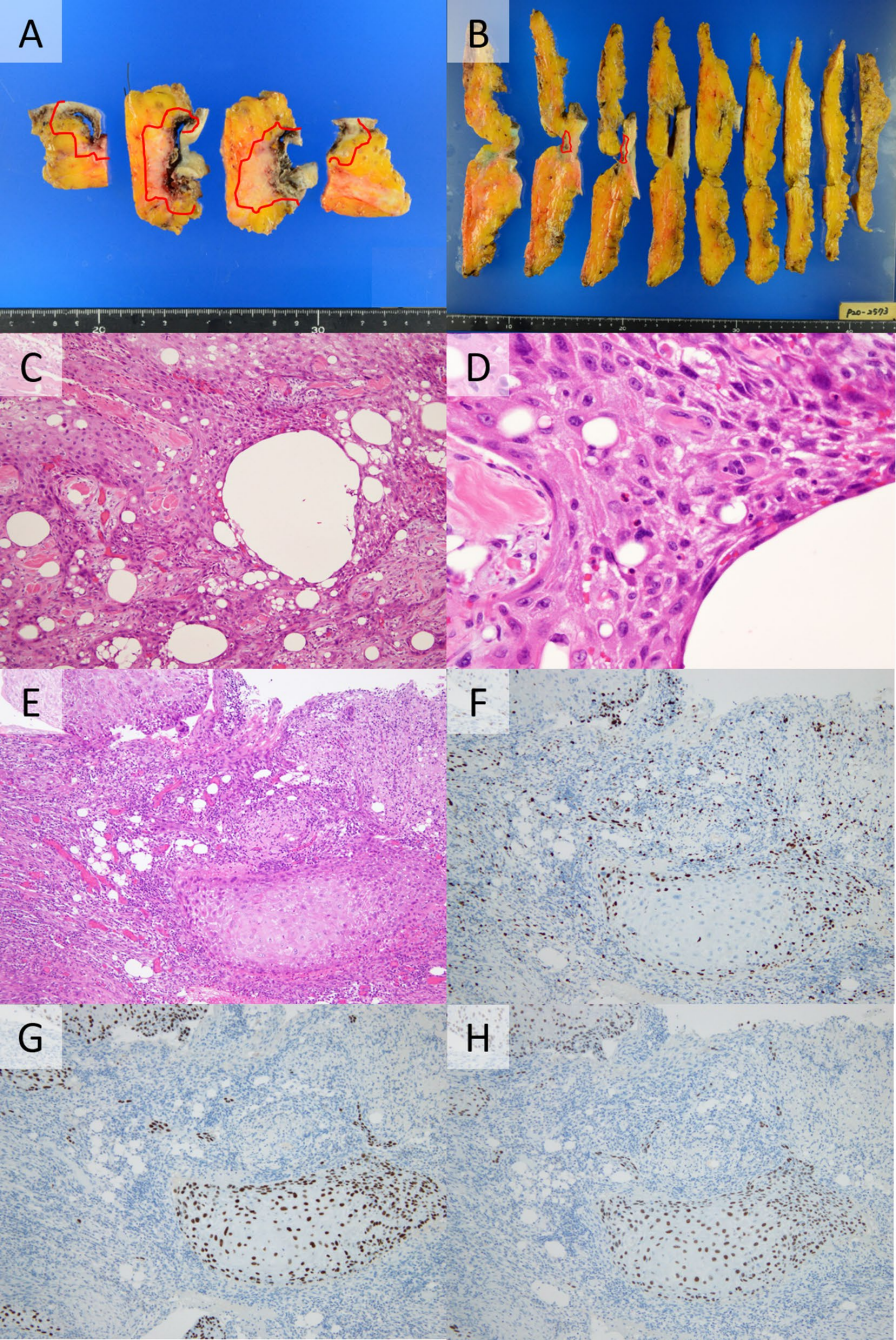
Histopathological images. **A** Partial mastectomy **B** Total mastectomy **C** Hematoxylin–eosin staining (weak expansion) **D** Hematoxylin–eosin staining (strong expansion) **E** Hematoxylin–eosin staining **F** Ki67 10% **G** p40 positive **H** p63 positive. **A**, **B** Squamous cell carcinoma was found in the red line area. **C** Lots of indistinct cavities of foreign particles, probably silicon, surrounding squamous cell carcinoma. **D** Intercellular bridges are observed

The gross appearance of the breast tumor was not distinctive and with cystic degeneration and skin ulcer. The cut surface showed white-to-greyish. Microscopically, the cyst wall was lined by atypical squamous epithelial cells with varying degrees of nuclear atypia and prominent keratinization, and in the deep layer, these atypical cells infiltrated into the surrounding tissues and occasionally showed a spindle shape. In the surrounding mammary tissue, numerous vacuolated structures of different sizes were observed accompanied with inflammatory cell infiltration, and fibrosis. Immunohistochemically, these atypical cells were positive for p40 and p63 (Fig. 3). Estrogen receptor, progesterone receptor, and human epidermal growth factor receptor 2 (HER2) were negative in tumor cells. The histopathological diagnosis was squamous cell carcinoma, pT3 pN0 pM0 Stage IIB.

**Fig. 4:**
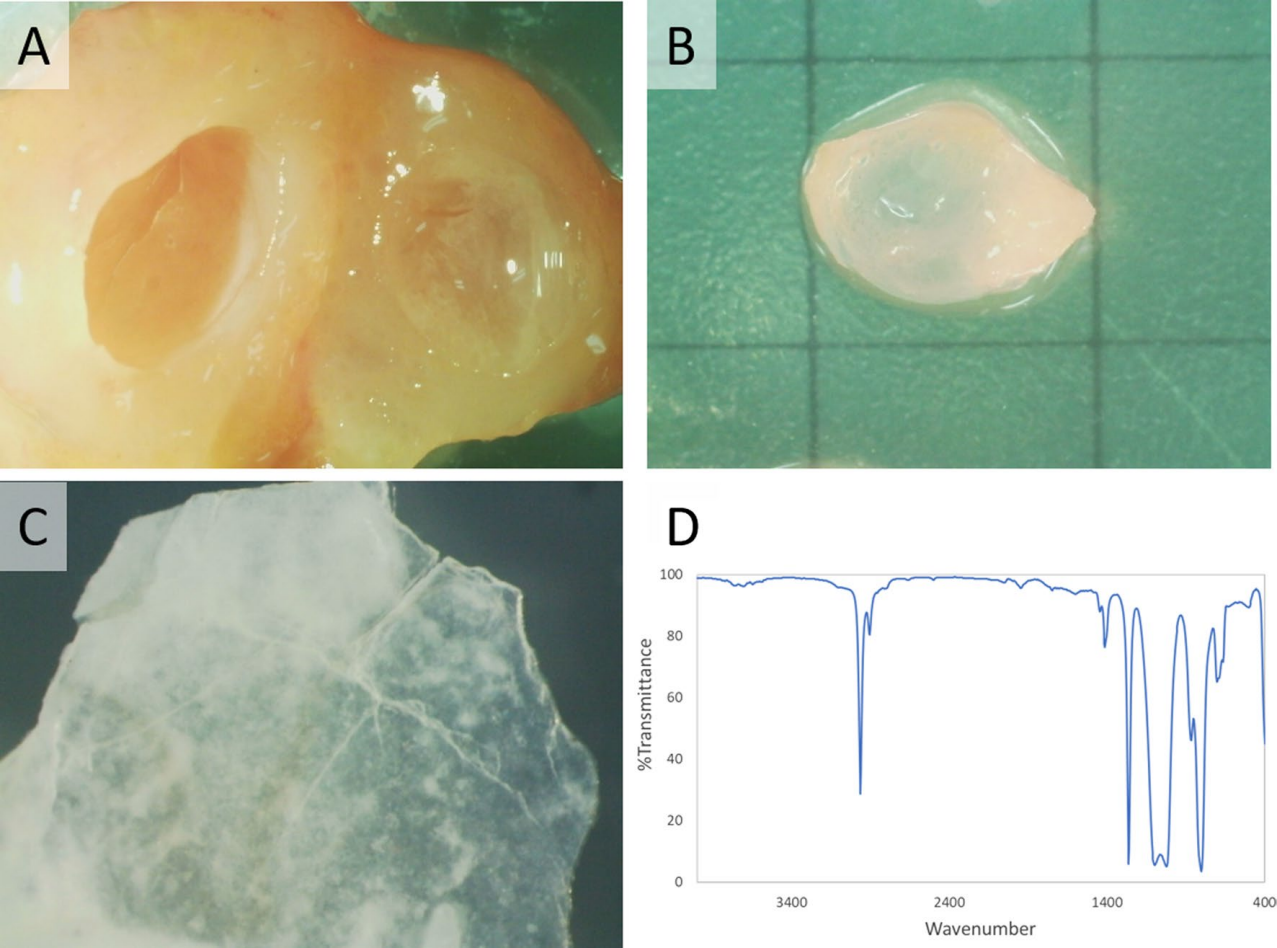
Analyses of the multiple cysts and their contents. **A** Multiple cysts with high viscous liquid contents were observed in the removed mammary tissue. **B**, **C** Remained cyst wall after protein/lipid digestion. Thin hard shell remained when cysts were placed in a protein/lipid-digesting solution for 2 weeks. **D** Liquid contents in the cysts were isolated and analyzed by Fourier transform infrared spectrophotometer at the Chemicals Evaluation and Research Institute, Japan

Multiple cysts diameters from 0.2 to 1.5 cm were observed in the removed mammary tissue. Thin hard shells remained after surrounding fatty tissues were digested from the cysts with protein/lipid-digesting solution for 2 weeks. The liquid contents in the cysts were isolated and analyzed by Fourier transform infrared spectrophotometer at the Chemicals Evaluation and Research Institute, Japan. The strong absorption bands in the mid-infrared spectrum range, at 1261.4, 1094.7–1022.5, and 800.9 cm-1 indicated that the liquid contents were polydimethylsiloxane (e.g., silicone oil) (Fig. 4).

## Discussion

In Japan, breast augmentation began to be actively practiced in the late 1950s and 1960s. Around 1965, however, due to the complications associated with the injection method, it was replaced by the use of a silicone bag prosthesis, in which a foreign body is encapsulated in a silicone bag. Although, the leakage of the contents from damaged bags was initially problematic, increasing the durability of soft cohesive bags improved the silicone bag insertion method. Currently, bag insertion is the mainstream breast augmentation method.

Injecting liquid silicone or leakage into the breast leads to various complications. The most common complications are silicone mastitis and silicone sarcoma [11–13]. Silicone dissemination to surrounding tissues may lead to autoimmune/inflammatory syndrome induced by adjuvants (ASIA) also called human adjuvant disease (HAD) have also been noted as systemic complications [14].

Breast augmentation-associated cancer is difficult to diagnose preoperatively from the presence of foreign bodies, especially in the case of breast augmentation with direct injection of a foreign body. The injected foreign body itself or induced mastitis makes it harder to detect cancer.

In this case, visualization of the diseased part was not easy with ultrasonography. The echo beam was not able to penetrate both breasts. On the other hand, MRI can identify foreign bodies in the breast. Contrast-enhanced MRI was useful in distinguishing injected foreign bodies from breast cancer [15, 16].

It is reported that the incidence of breast cancer after breast augmentation is 37.5% for silicone implants, 10.4% for paraffin implants, 10.4% for silicone bags, and 0.02% for fat implants. There is no clear evidence that indicates the incidence depending on the type of filling materials, though [1]. In this case, the injected material was identified as the silicone from the sample taken at the time of surgery.

Although various histological types of breast cancer occurring after breast augmentation have been reported, most of them are adenocarcinomas, and squamous cell carcinomas are uncommon [1–10]. Particularly primary squamous cell carcinoma of the breast is extremely rare. According to the World Health Organization (WHO) classification of breast tumors in 2003, the diagnosis criteria of primary squamous cell carcinoma are the following: (1) a tumor origin that is independent of the overlying skin and nipple or adnexal elements; (2) more than 90% of the tumor must be squamous; and (3) other sites of primary squamous cell carcinoma must be excluded [17]. All the conditions were satisfied in this case to diagnose as primary squamous cell carcinoma in the breast.

Patients diagnosed with squamous cell carcinoma can be classified into two main types: the mixed type, which is a mixture of ductal carcinoma and squamous cell carcinoma, and the pure type, which is formed purely from squamous cell carcinoma [18].

Azzopardi et al. further classified breast squamous cell carcinoma into four types: (1) adenocarcinoma with varying degrees of squamous metaplasia, (2) squamous cell carcinoma with extensive spindle cell metaplasia and/or marked desmoplasia, (3) squamous cell carcinoma arising in cystosarcoma phyllodes, and (4) pure squamous cell carcinoma [19].

This case was considered a pure squamous cell carcinoma on histopathological examination. Estrogen receptor and progesterone receptor were not detected, and there were no areas suggestive of transformation from adenocarcinoma to squamous cell carcinoma. The high degree of inflammatory cell infiltration suggested the development of squamous cell carcinoma due to chronic inflammation.

There are several theories on the histogenesis of squamous cell carcinoma of the breast, such as squamous epithelialization of glandular tissue or adenocarcinoma transforming into squamous cell carcinoma.

Cases of squamous cell growth and squamous cell carcinoma arising from the surface of the implant capsule have also been reported [20, 21], and it is possible that squamous cell carcinoma was derived from fibrosis encapsulating the peri-silicone vacuole as a chronic inflammatory change after liquid silicone injection.

The management of breast squamous cell carcinoma has not been standardized due to the insufficiency of cases. Surgery is the cornerstone of the primary treatment of breast squamous cell carcinoma. In most patients, breast-conserving surgery is not possible due to the advanced stage of the disease.

Although squamous cell carcinomas are often radiosensitive, several studies reported poor responses to radiation therapy of breast squamous cell carcinomas, and the recurrence-free rates are similar to those of patients not receiving radiation therapy. [22, 23].

Neoadjuvant and adjuvant chemotherapies can be applied for breast squamous cell carcinoma with combinations of 5-fluorouracil, cisplatin, paclitaxel, cisplatin, and paclitaxel [24]. Several studies failed to show a significant benefit from neoadjuvant chemotherapy [25], thus there is still no established treatment. As in this case, chemotherapy for breast squamous cell carcinoma is often given with the same regimen as for triple-negative breast cancer.

## Conclusion

Liquid silicone injection is not a current breast augmentation option, but injected silicone can lead to cancerous tumor development after years. This case illustrates how non-physiological substances can cause unexpected biological reactions that are difficult to diagnose with routine medical examinations. It is essential to accumulate data from more cases of breast squamous cell carcinoma to establish efficient treatment for this disease.

## Data Availability

The data sets used and/or analyzed during the current study are available from the corresponding author on reasonable request.

## Abbreviations

SSC: Squamous cell carcinoma
OSNA: One step nucleic acid amplification
HER2: Human epidermal growth factor receptor 2
CT: Computed tomography
MRI: Magnetic resonance imaging
